# Reverse Spillover Dominating CO Adsorption on Single Cobalt Atoms in Graphene Divacancies

**DOI:** 10.1021/acs.jpcc.4c07088

**Published:** 2024-12-25

**Authors:** Francesco Armillotta, Pardis Naderasli, Valeria Chesnyak, Harald Brune

**Affiliations:** † Ecole Polytechnique Fédérale de Lausanne (EPFL), Station 3, CH-1015 Lausanne, Switzerland; ‡ Physics Department, University of Trieste, via A.Valerio 2, 34127 Trieste, Italy; § CNR-Istituto Officina dei Materiali (IOM), Strada Statale 14, km 163.5, 34129 Trieste, Italy

## Abstract

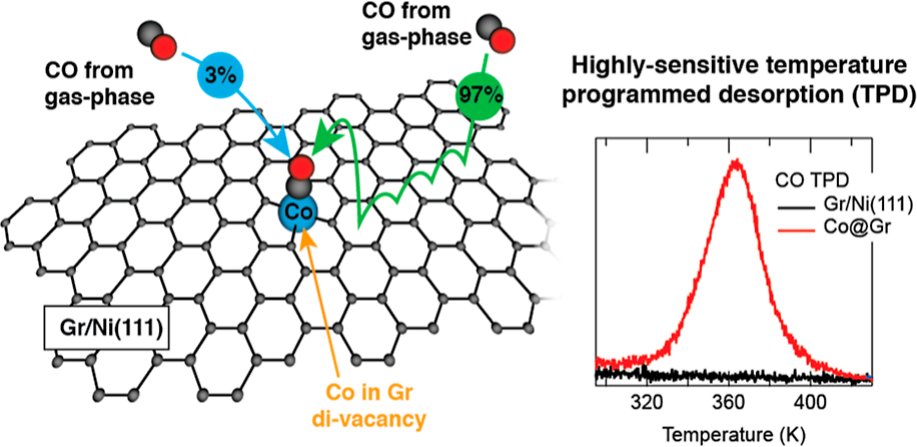

The kinetics of molecular adsorption and desorption can unveil the details of the adsorption potential that impact, for instance, the overall sticking probability. This information is of particular importance for catalysis and gas sensing. We investigate the room-temperature CO adsorption on a model single-atom catalyst consisting of single Co atoms trapped in graphene (Gr) double carbon vacancies during Gr growth by chemical vapor deposition (CVD) on Ni(111). The study is conducted by combining a thermal desorption spectroscopy (TDS) instrument that allows the study of systems with a very low surface density of active sites, of the order of 10^–3^ monolayers (MLs) with variable-temperature scanning tunneling microscopy (VT-STM). Our findings show that CO adsorption onto the single Co atoms occurs mainly (up to 97%) through a reverse spillover mechanism, rather than through direct impingement from the gas phase. This mechanism involves CO physisorption and diffusion on pristine Gr, followed by lateral adsorption onto Co atoms. The reverse spillover channel effectively increases the sticking probability, by up to 2 orders of magnitude, compared with direct impingement. We use kinetic models to determine the relevant energies, such as the diffusion barrier for CO on Gr (68 ± 15 meV), the energy barrier for lateral CO adsorption on Co (174 ± 2 meV), and the chemisorption energy of CO on Co (0.97 ± 0.02 eV).

## Introduction

Single-atom catalysis (SAC) represents a cutting-edge frontier in heterogeneous catalysis, where individual atoms serve as active sites for chemical reactions.^1,2^ The high level of dispersion not only minimizes the consumption of raw material but also enables the creation of well-defined active centers with specific coordination environments, making SAC comparable to homogeneous catalysis. The reduced coordination of single atoms generally leads to a narrower energy spread of relevant orbitals, enhancing selectivity and creating uniformity among active sites. Additionally, reaction intermediate species adsorbed on single atoms have been shown to exhibit different scaling relations compared to those on extended surfaces, so far widely investigated, which opens the possibility for unexpected reaction pathways.^3^

To preserve the coordination geometry and prevent aggregation, single atoms in SACs must be stabilized on a supporting material. A promising approach is to embed single atoms in a rigid matrix. Graphene, for instance, has been suggested as an optimal support due to its high conductivity, surface to volume ratio, and abundant carbon availability.^4−9^ Single atoms can form strong covalent bonds with Gr carbon atoms, becoming both thermally and chemically stable. Single atoms dispersed in Gr have already proven to be a viable alternative to traditional catalysts, demonstrating high performances in several technologically important reactions, such as CO_2_ reduction, CO oxidation, benzene oxidation, oxygen evolution and reduction reactions, and methane conversion.^10−15^

Despite enormous scientific and technological effort, there are still too few experimental insights into the fundamental reaction dynamics at the atom scale. The measurement of thermodynamic and kinetic quantities and their comparison with the numerous theoretical predictions are crucial for advancing the understanding and engineering of SACs. Without this step, the predictive power of calculations remains quite limited.^16^ To bridge this gap, it is essential to study model systems that facilitate experimental investigation. The surface science offers a powerful approach, providing well-established high-resolution techniques and a nearly contamination-free environment, made possible by the use of ultra-high vacuum (UHV) chambers.^17^

In this manuscript, we investigate the CO chemisorption kinetics on a model SAC, where single Co atoms are intentionally trapped in Gr double carbon vacancies (Co@Gr) during Gr growth by chemical vapor deposition (CVD) on Ni(111).^18^ Previous studies have demonstrated that CO can bind stably to Co@Gr even at room temperature.^19^ The bonding stability arises from optimal electronic configuration of the Co 3d_*xz*,*yz*_ orbitals, which are primarily responsible for CO binding through backdonation to the CO empty π* orbitals. The Co 3d_*xz*,*yz*_ electronic structure results from the lateral hybridization of Co with the Gr π band, which is n-doped on Ni(111), leading to half-filled orbitals close to the Fermi level.

Our findings reveal that the CO sticking probability is significantly increased—by 2 orders of magnitude—by the presence of an additional adsorption channel beyond direct impingement from the gas phase. This mechanism, known as reverse spillover, involves CO physisorption and diffusion on pristine Gr, followed by lateral adsorption onto the single Co atoms. The insight gained from this study could be valuable in the fields of heterogeneous catalysis and gas sensing. In the latter case, Gr has also been proposed as an optimal support material due to its his large surface area to volume ratio, high conductivity, high crystallinity, and suitability for four-probe measurements on a single-crystal device.^20,21^

## Experimental and Theoretical Methods

Experiments are carried out in a UHV chamber with a base pressure below 1 × 10^–10^ mbar.^22,23^ The chamber is equipped with a home-built “Beetle”-type variable-temperature scanning tunneling microscope, an e-beam evaporator (triple evaporator EFM 3T, Focus), an ion sputter gun (SPECS IQE 12/38), ethylene gas-lines for Gr growth, and the thermal desorption spectroscopy (TDS) device described in more detail below. The sample can be heated by filament radiation and electron bombardment and cooled with a flux cryostat down to 30 K by LHe and to 130 K by LN_2_. The temperature is measured by a Ni–Cr/Ni–Al thermocouple (K-type) whose wires are spot-welded onto the brim of the crystal. The reference thermocouple junction is placed in a thermally stabilized preamplifier (H. Schlichting, Pureions). The power supplies for both the filament and the high-voltage for electron bombardment are controlled using two interdependent PID loops (Eurotherm iTools). The absolute temperature is calibrated with the Xe multilayer desorption peak located at 60 K.^24^

### Sniffer

In the same UHV chamber, reaction gas dosing and detection are performed using a home-built system called a Sniffer. This system encloses the sample surface within a small desorption volume that is separately pumped. A commercial quadrupole mass spectrometer (QMA 200 Pfeiffer Vacuum) is positioned at the end of this detection volume. The detection limit of this setup is as low as 3 × 10^–5^ ML/s [1 ML is defined by one molecule per Ni(111) surface atom]. The ionizer is modified to prevent direct line-of-sight between the hot filament and the sample. The probing gas (^13^C^16^O in our case) is introduced directly into the Sniffer volume using electro-valves (Parker, series 99), which are activated by a rectangular voltage pulse of typically 1–4 μs duration and 28 V amplitude, allowing partial opening of the valve.^25^ The Sniffer is an uystem used by Bonanni et al.^25,26^ In our case, a heated quartz tube defines the internal walls of the desorption volume, and a linear travel motion facilitates an easy approach to the sample. To calibrate the CO dosing (monitored by the Sniffer itself) and the desorption signal in terms of absolute coverage, the procedure described in Supporting Discussion 1 is followed. Unless specified otherwise, all TDS experiments reported in this article are conducted with a heating rate of 1 K/s.

### Sample Cleaning and Growth

The Ni(111) surface was cleaned through multiple cycles of Ar^+^ sputtering at 1.0 keV (1 μA/cm^2^, 300 K) followed by annealing at 873 K for 20 min. Gr was grown on Ni(111) using CVD by dosing ethylene (5 × 10^–7^ mbar) at a sample temperature of 833 K for 1 h.^27^ The completion of a Gr layer was confirmed by the absence of any signal in the CO TDS spectra between 250 and 430 K, indicating that no clean Ni or Ni_2_C patches were present.^28−30^ Co atoms were incorporated into graphene during CVD growth.^18^ The clean Ni(111) sample was heated to 833 K and exposed to both ethylene (5 × 10^–7^ mbar) and a constant Co flux (0.003 ML/min) from an e-beam evaporator equipped with flux control. After 30 min, the Co flux was stopped, and Gr growth was continued for an additional 30 min, before closing the ethylene and cooling the sample.

## Results and Discussion

### CO Adsorption on Gr/Ni(111) and Co@Gr/Gr/Ni(111)

**Figure 1:**
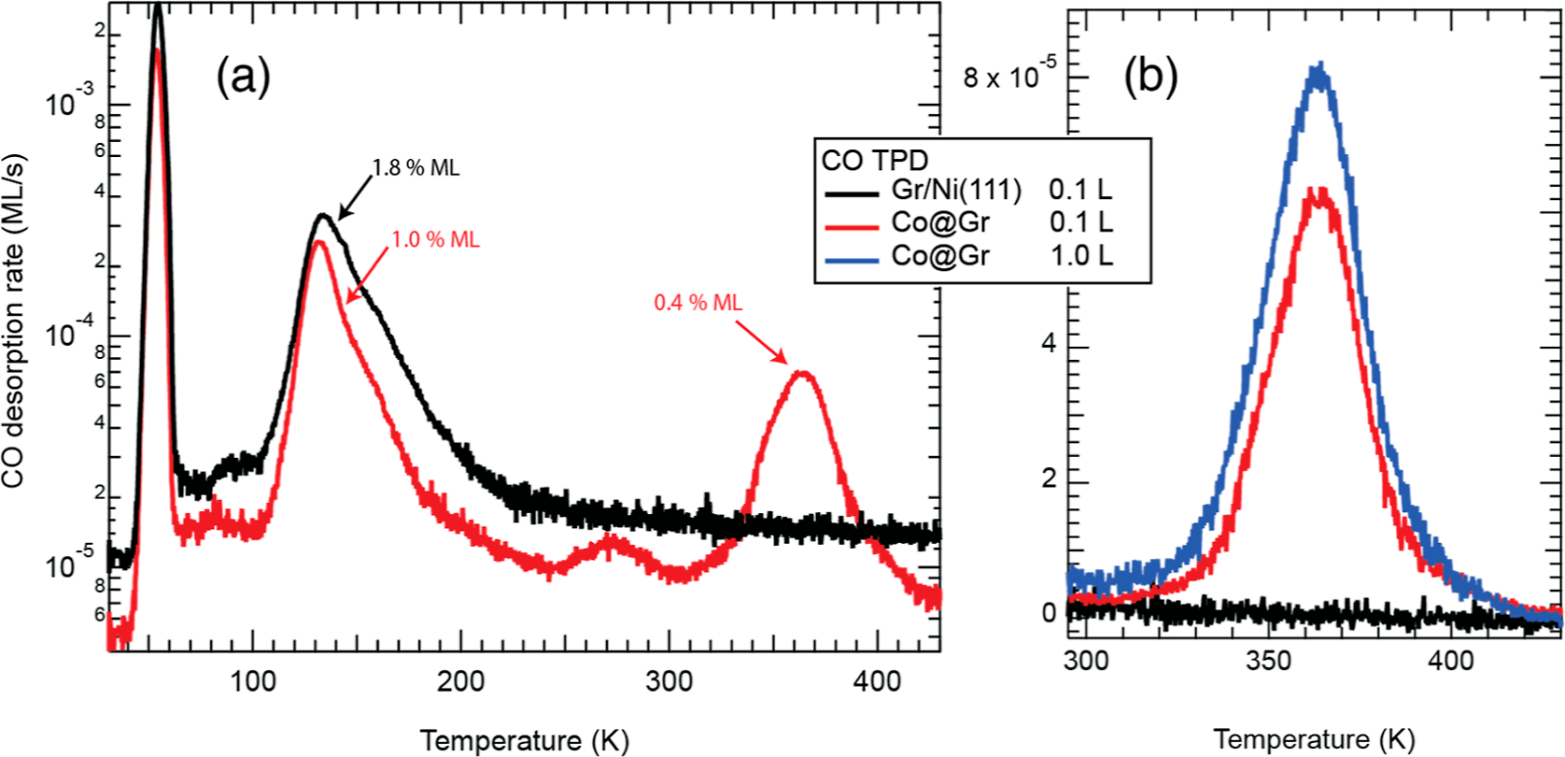
CO TDS spectra of the Gr/Ni(111) sample with and without Co incorporation in Gr double carbon vacancies, reported in the (a) semilogarithmic and (b) linear scale. The TDS spectra are acquired after dosing CO at 30 K. The heating rate is 1 K/s.

Figure 1a (black curve) shows the CO TDS spectrum of Gr/Ni(111), after dosing with 0.1 L at 30 K. TDS is performed immediately after (∼1 h) the sample growth and by keeping the latter in UHV. The most intense desorption peak is found at 53 K. The corresponding desorption energy (*E*_d_) and attempt frequency (ν_d_) can be found by fitting the low-temperature part of the peak (Figure S1). The fit gives *E*_d_ = 141 ± 1 meV and ν_d_ = (7.1 ± 0.4) × 10^12^ Hz. The saturation coverage of this peak is 0.3 ML. Therefore, it is attributed to the physisorbed CO onto pristine Gr. We further conclude from our TDS that Gr entirely covers the sample surface, since remaining clean Ni or Ni_2_C patches would cause CO to desorb at much higher temperatures, between 300 and 430 K, where our spectra are completely flat.^28−30^ STM images also confirm the absence of any clean Ni or Ni_2_C patches (Figure S2).

Another peak is visible at 140 K on Gr/Ni(111), with a saturation coverage of 1.3% ML. We believe it corresponds to CO desorbing from Ni-occupied carbon vacancies in Gr (Ni@Gr), which are also present when graphene is grown at low temperature on Ni(111).^31,32^Figure S2 presents an STM image acquired on Gr/Ni(111), showing bright protrusions attributed to Ni@Gr based on the literature, with a coverage of 1.6% ML (note that the sample is homogeneous on the mesoscopic scale but not on the nanoscopic scale). However, the precise attribution of the 140 K peak is beyond the scope of this paper, does not affect its results, and will be addressed in a subsequent work.

**Figure 2:**
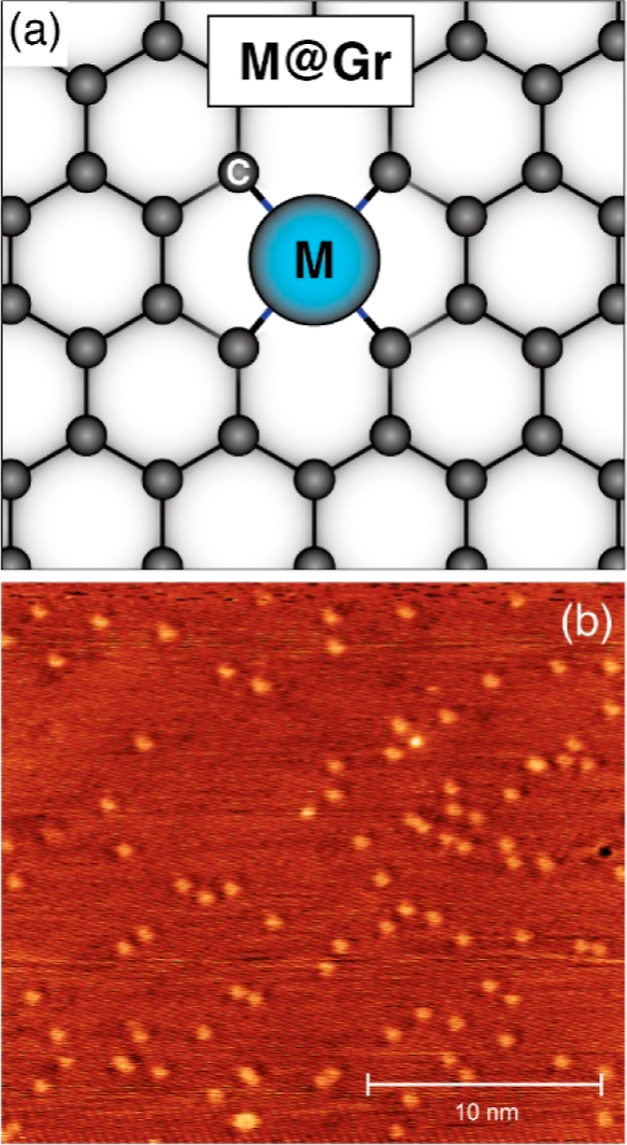
(a) Schematic model of the atomic arrangement of a single metal (M) atom in a Gr double carbon vacancy (M@Gr) and (b) room-temperature STM image of Co@Gr/Gr/Ni(111) (*I* = 300 pA, *V*_bias_ = +2 V). The image shows a region where the graphene grows pseudomorphically, without displaying any moiré pattern. The bright protrusions (0.8% ML) are attributed to Ni@Gr and Co@Gr.^18^

When Gr is grown by CVD with the coevaporation of Co atoms, these atoms can occupy double carbon vacancies in the Gr lattice (Co@Gr).^18^ The Co atoms that are not trapped (90%) undergo dissolution into the crystal bulk. A schematic model of the Co@Gr atomic arrangement is provided in Figure 2, together with an STM image of the pristine Co@Gr/Gr/Ni(111) sample.

A TDS spectrum with parameters identical to the one just discussed has been acquired and plotted in Figure 1a. A new feature rises at 360 K, attributable to CO binding to Co@Gr (Figure 1a,b).^19^ According to the Redhead approximation for first-order desorption (Figure S3),^33^*the CO binding energy on Co@Gr is* 0.97 eV, supposing an attempt frequency of 1.0 × 10^13^ Hz. Another feature, also attributable to the presence of Co, can be observed at 270 K, with a maximum saturation coverage on the order of 10^–4^ ML, thus making its exact attribution very challenging. In what follows we therefore focus on the 360 K peak. Small vertical offsets in the spectra are due to slight differences in the chamber vacuum between different experiments.

After dosing 0.1 L of CO, the coverage of CO desorbing from Co@Gr (θ) is 4.1 × 10^–3^ ML. Increasing the dose to 1 L causes the saturation of the Co adsorption sites: θ_Sat_ = 5.8 × 10^–3^ ML (Figure 1b). Thus, the latter value also corresponds to the total Co@Gr amount, and we conclude that, after 0.1 L, the CO molecules already occupy 70% of the sites. The Co density is in good agreement with previous STM data, which reported a density of 5 × 10^–3^ ML.^18^ Assuming that the 140 K peak is related to Ni@Gr, we have a total density of Co,Ni@Gr single atoms of 1.4% ML, not far from what we observe in the STM of Figure 2b, which gives 0.8% ML. The difference is due to the large inhomogeneity of the density of single atoms. In order to achieve a statistically significant density estimate from STM, one would need to image several hundreds of macroscopically distant sample areas.

We can estimate the amount of CO adsorbing directly from the gas phase onto the single Co atoms (θ^gp^) by using the low-coverage approximation of the Langmuir isotherm
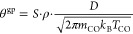
1where *S* is the sticking probability, ρ is the Co@Gr surface fractional area (ρ = 0.008, by attributing a radius of 1.5 Å to Co@Gr^34^), *D* [Pa·s] is the CO dosage corresponding to 0.1 L, and *m*_CO_ = 29 amu and *T*_CO_ = 300 K are, respectively, the mass and the temperature of the isotopically labeled ^13^C^16^O that we dosed. The maximum value for θ^gp^ can be obtained by setting *S* = 1 and is equal to θ^gp^ = 1.2 × 10^–4^ ML. Remarkably, θ^gp^ is less than 3% of the CO amount we measured. Hence, there must be an additional adsorption channel, besides direct impingement from the gas phase, accounting for the remaining 97% of CO adsorption.

**Figure 3:**
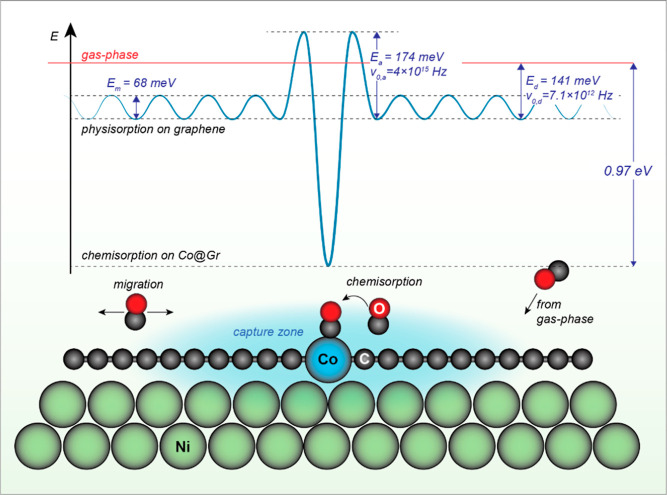
Side view of the sample and kinetic pathway for CO adsorption, superimposed with an energy diagram. The CO molecule can adsorb by direct impingement from the gas phase or laterally from Gr, following physisorption and diffusion on Gr.

The most plausible alternative pathway is represented by the adsorption of CO on pristine Gr, its diffusion, and subsequent lateral attachment to Co (reverse spillover). A side view of the sample and kinetic pathway is shown in Figure 3, superimposed with an energy diagram. The reverse spillover adsorption involves two energy barriers: the one accounting for CO migration on pristine Gr (*E*_m_) and the one that CO has to overcome in order to leave Gr and bind to Co (*E*_a_).

**Figure 4:**
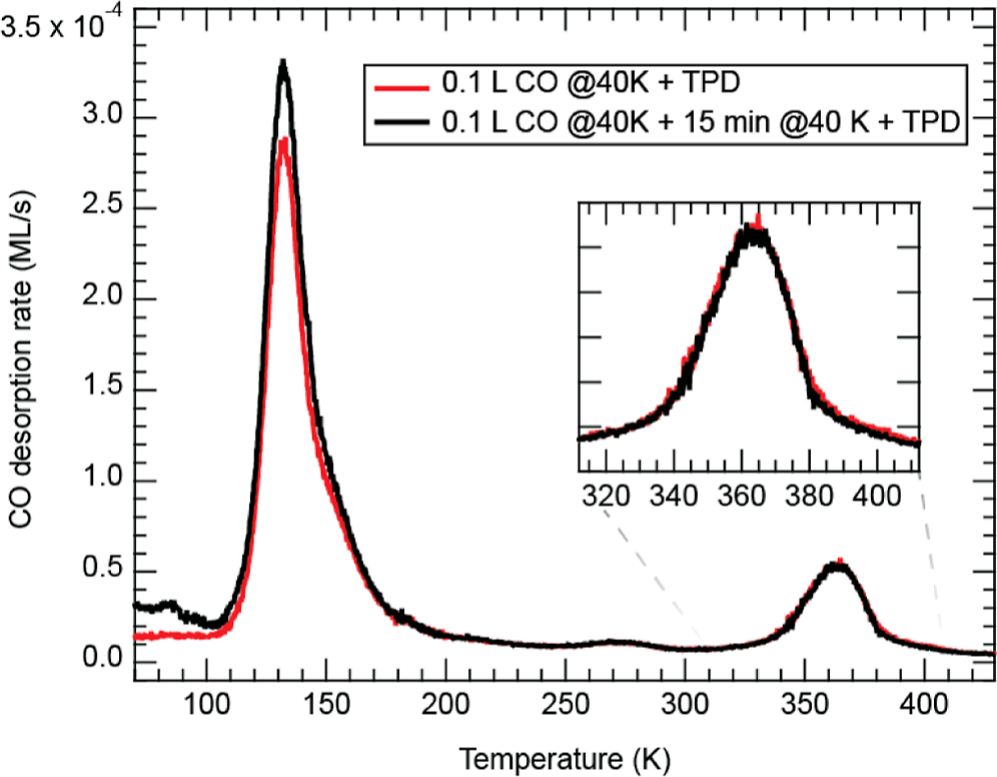
TDS spectra after exposure of the Co@Gr/Gr/Ni(111) sample to 0.1 L CO at 40 K. One spectrum (red curve) has been acquired immediately after exposure and the other (black curve) 15 min after it. CO adsorbed on Gr is mobile at 40 K. The peak at 360 K remains unchanged, suggesting the presence of an activation barrier (*E*_a_), too high for CO molecules to attach to Co@Gr at 40 K. Therefore, the CO molecules can only populate Co when the temperature is increased during the TDS run.

To get a first estimate on *E*_a_ and *E*_m_, we acquired two TDS spectra in identical conditions, except that in one case we waited 15 min at the dosing temperature (*T*_dos_ = 40 K) before starting the measurement (Figure 4). The waiting time has been chosen to be greater than (three times) the time necessary to acquire a TDS spectrum. The waiting temperature has been chosen in such a way that the majority of CO molecules (98.9%) have not desorbed from pristine Gr after 15 min. There are three possible scenarios regarding the final amount of CO on Co@Gr that will be measured:

First, if *E*_a_ = 0, all Co@Gr sites would soon saturate at 40 K. We can already exclude this scenario, since we already observed only 70% of saturation with the same amount of CO dosing. Second, if *E*_a_ is finite and sufficiently low, during the waiting time the CO molecules will continue to diffuse and bind to the Co sites. Thus, after waiting 15 min, the final coverage should increase. Third, if *E*_a_ is finite and sufficiently high, no extra adsorption will occur at 40 K. In this case, CO can only chemisorb *during* the TDS measurement. In this case, desorption from pristine Gr and lateral attachment to Co will be competing processes.

Figure 4 shows no increase in the CO–Co@Gr coverage after waiting 15 min at 40 K, meaning that scenario 3 is verified. We can provide a lower bound estimation for *E*_a_ by setting
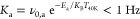
2or
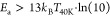
3with *K*_a_ being the adsorption rate and assuming ν_0,a_ = 10^13^ Hz. Thus, we get that *E*_a_ must be larger than 100 meV. On the other hand, *E*_a_ cannot be too high, otherwise all CO molecules will desorb from Gr, during TDS, before they have the chance to laterally attach to Co. Following a similar reasoning and considering that CO is completely desorbed from Gr at about 70 K (Figure S1)
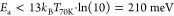
4Thus
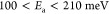
5In Figure 4, we can also observe that after waiting 15 min the peak at 140 K is increased. If, as we proposed, this peak corresponds to CO adsorbed on Ni@Gr, it would mean that the adsorption mechanism is similar (e.g., reverse spillover) and that the corresponding activation barrier is lower than that in the case of Co@Gr. It also witnesses the fact that CO can actually diffuse at 40 K, and following a similar reasoning of eq 2, we can say that *E*_m_ must be less than 100 meV.

### Kinetic Model for CO Adsorption on Co@Gr

We now provide a more precise estimation for *E*_a_. We start by writing down a schematic kinetic model for our system
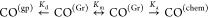
6where we label CO^(gp)^, CO^(Gr)^, and CO^(chem)^ as the CO species in the gas phase, physisorbed on Gr, and chemisorbed on Co@Gr, respectively. *K*_d_, *K*_m_, and *K*_a_ are the corresponding rates of desorption of CO from Gr, migration on Gr, and lateral attachment to Co@Gr, respectively. *K*_m_ is linked to *E*_m_ via the Arrhenius equation
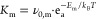
7

The corresponding rate equations are
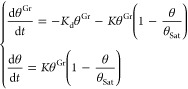
8where, θ^Gr^ is the coverage of CO on pristine Gr at a given time. *K* is the CO chemisorption rate on Co. *K*, in general, depends on both the migration (*K*_m_) and adsorption (*K*_a_) rates
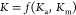
9

We proceed further by determining *K*_m_ in eq 9 and then solve the differential equations in 8 and find *E*_a_.

### CO Migration on Pristine Gr

**Figure 5:**
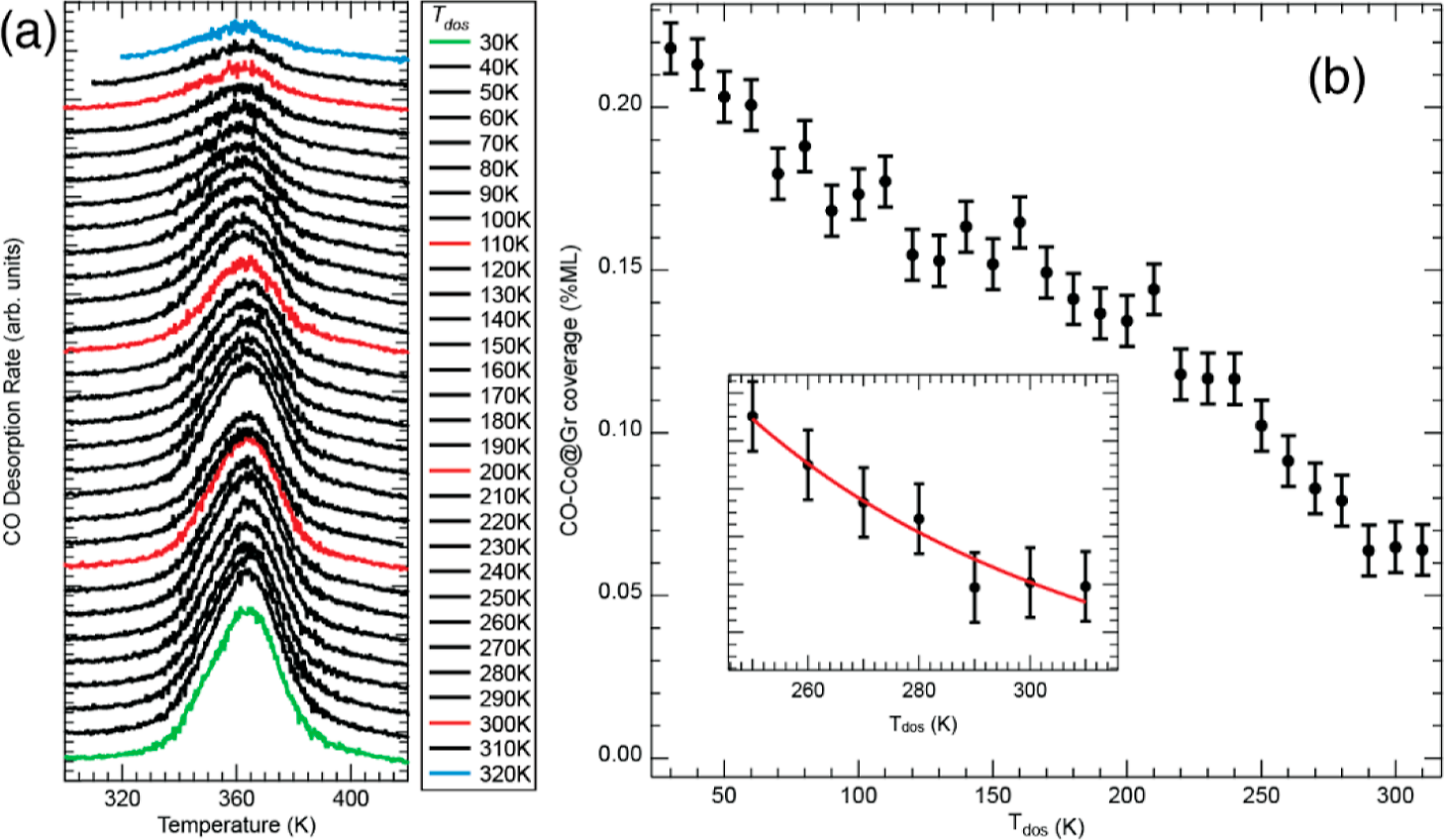
(a) TDS spectra after exposure of the Co@Gr/Gr/Ni(111) sample to 0.10 L CO at temperature *T*_dos_ and (b) CO coverage on Co@Gr as a function of *T*_dos_ measured by integrating the area of the peak at 360 K in panel (a). The inset shows the experimental data (black dots) together with the fit (red solid line). The fit was done by using the expression for the CO coverage in eq 15. The model implies the attribution of a capture area for CO around Co@Gr. The capture area size depends on the average CO residence time on graphene and, ultimately, on the sample temperature.

*E*_m_ can be extracted from the temperature dependence of the CO adsorption probability on Co@Gr. According to our picture, we can attribute a capture area *[A*_C_(*T*)] to Co@Gr, in which the CO molecules can diffuse and attach to the metal center. In this way, the geometrical factor ρ in eq 1 will increase by
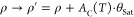
10*A*_C_(*T*) will depend on the mean square diffusion length (*Χ*_S_) that CO has on pristine Gr, which is a function of *E*_m_, and decreases by increasing the sample temperature. Hence, we dosed 0.10 L of CO at a temperature *T*_dos_ in the range between 30 and 320 K, in steps of 10 K. After each dosing, we run a TPD acquisition starting from *T*_dos_ until 430 K. The spectra are shown in Figure 5a. To extrapolate the corresponding CO coverage, the spectra have been integrated between 320 and 410 K, by considering a linear background, converted to a surface density, and plotted as a function of *T*_dos_ as shown in Figure 5b. As expected, the final CO coverage on Co@Gr diminishes with an increase in the dosing temperature *T*_dos_.

To describe the temperature dependence of the sticking probability and extract the information about *E*_m_, we can use the results of Henry and Chapon describing a similar system, cadmium atoms adsorption on NaCl-supported gold particles.^35^ Similarly to our case, the Cd atoms can attach either by direct impingement or by reverse spillover after diffusion on NaCl. The contribution of the latter channel can be calculated by looking at the adsorbate radial surface density *n*(*r*, *t*) in the proximity of a gold particle (or Co@Gr in our case) at *r* = 0. The evolution of *n*(*r*, *t*) is given by
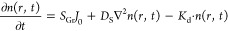
11

*S*_Gr_ is the CO sticking probability on Gr, *J*_0_ is the CO incident flux on the surface, and *D*_S_ is the diffusion coefficient. Referring to our system, on the right side of the equation, the first term considers CO impingement from the gas phase; the second term, CO random-walk diffusion on Gr; and the last term, CO desorption from Gr. At equilibrium, the solution can be found by imposing . As a boundary condition, we set *n*(*R*) = 0, where *R* is the distance at which the CO can bind to Co without further migration. In particular, the latter condition underlies that CO has a high chance to overcome the activation barrier *E*_a_, which is true if *K*_a_ ≫ 1 (*T* ≫ 70 K). Also, in our case, Co can only bind one CO at a time; thus, the hypothesis for the solution above is valid if the capture regions of the different Co centers do not overlap each other. This condition can be expressed by writing
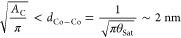
12where the latter term is the mean Co–Co distance. With these hypotheses, eq 11 can be solved analytically, and the capture area *A*_C_ around the cluster is obtained by calculating the flux reaching the Co atom by surface diffusion^35^
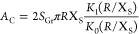
13

*K*_*n*_ is the *n*-order Bessel function of the second kind, and *Χ*_S_ is given by
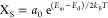
14

In eq 14, we make the hypothesis that ν_0,m_ ≃ ν_0,d_. The curve of Figure 5b can now be fitted by using eq 1 and by substituting ρ with ρ′
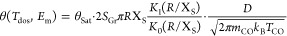
15

Here, we ignore adsorption by direct impingement since, as we have seen earlier, it contributes less than 3%. Equations 1 and 15 are low-coverage approximations of the Langmuir isotherm and thus to stay in the regime of linearity between θ and CO dose, we only fit the points in Figure 5b for which θ < 0.3θ_Sat_. The best fitting curve is shown in the inset of Figure 5b (solid red line). The fit result gives *E*_d_ – *E*_m_ = 73 ± 14 meV and *S*_Gr_ = 0.26 ± 0.11 (*S*_Gr_ is an average over the temperature range). Thus, *E*_m_ = 68 ± 15 meV. *R* has been fixed to 1.5 Å, and its variation by 0.1 Å affects *E*_d_ – *E*_m_ by 0.3 meV and *S*_Gr_ by 0.01. The radius of the Co capture area at 250 K is 6 Å, validating the inequality in eq 12.

### Measurement of *E*_a_ and ν_0,a_

In order to solve the rate equations in (eq 8), we needed to find an explicit expression for eq 9. By knowing that *E*_a_ > 103 meV and *E*_m_ = 68 ± 15 meV, we can estimate the *K*_m_/*K*_a_ ratio in the temperature range in which we know CO adsorption on Co@Gr can occur, i.e., from 40 to 70 K
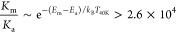
16
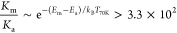
17Thus, eq 9 becomes *K* ≈ *K*_a_, this is because  means that overcoming the energy barrier *E*_a_ is the rate-determining step of the reverse spillover adsorption channel. Thus, we can rewrite eq 8 as
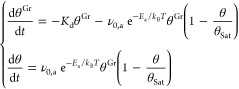
18

**Figure 6:**
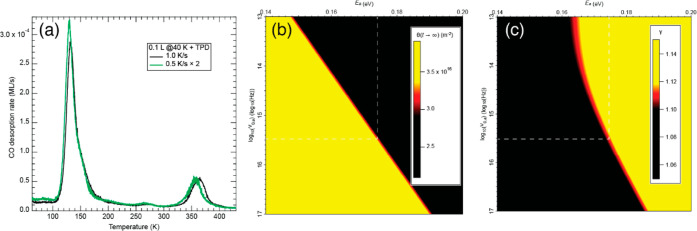
(a) TDS spectra after exposing the Co@Gr/Gr/Ni(111) sample to 0.10 L CO at 40 K. The heating rates are 0.5 K/s (green curve) and 1.0 K/s (black curve) and (b,c) false-color 2D maps showing the outcomes of the numerical simulation of the TDS spectra presented in panel (a). For each simulation, corresponding to a (*E*_a_, ν_0,a_) couple, we report in panel (b) the final CO on Co@Gr coverage (θ(*t* → ∞)) and γ in panel (c). The (*E*_a_, ν_0,a_) providing values for θ(*t* → ∞) and γ compatible with the experiment are shown in red.

The two unknown parameters in the equation above are *E*_a_ and ν_0,a_. In order to find them, we performed two identical TPD runs with different heating rates (0.5 and 1.0 K/s, after dosing 0.100 ± 1 L of CO at 40 K). The corresponding spectra are shown in Figure 6a. Being a first-order desorption, the peak shifts to a lower temperature by decreasing the heating rate. For better visual comparison, in Figure 6a, the curve taken at 0.5 K/s has been multiplied by a factor of 2. We find that decreasing the heating rate affects the final CO–Co@Gr coverage. Indeed, the coverage ratio (γ) is
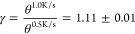
19

This means that having CO desorption from Gr and adsorption on Co@Gr as competitive processes, slower adsorption rates favor the first process, which is thus likely to possess a lower activation barrier. To find *E*_a_ and ν_0,a_, the two TDS runs have been simulated by numerical integration of eq 18. This has been done multiple times, by varying each time *E*_a_: 140 → 200 meV with steps of 0.12 meV and ν_0,a_: 10^13^ → 10^17^ Hz with steps of 0.08 log_10_(Hz). The initial conditions have been chosen by setting θ(*t* = 0) = 0, and θ^*Gr*^(*t* = 0) equal to the sum of the TDS peaks at 53 K (desorbing from Gr) and 360 K (desorbing from Co@Gr). In this way, we exclude all of the CO species attaching to the other Gr defects. The boundary conditions for *t* → ∞ are given by γ and by θ^1.0K/s^(*t* → ∞) = (3.1 ± 0.3) × 10^16^ m^–2^, where the maximal error in the latter case is given by the TDS calibration. In Figure 6b,c, the simulated γ and θ^1.0K/s^ values are plotted, for each (*E*_a_,ν_0,a_) pair, in false-color 2D maps. The areas in red are attributed to the (*E*_a_,ν_0,a_) pairs giving a value for γ or θ^1.0K/s^ compatible with the experimental data. The intersection of the possible solutions gives *E*_a_ = 174 ± 2 meV and ν_0,a_ = (4 ± 2) × 10^15^ Hz. As expected, since *E*_a_ > *E*_d_, slower desorption rates will favor desorption from Gr, and consequently a minor amount of CO will be found to bind the Co@Gr sites. Eventually, the value found for *E*_a_ is consistent with the rate-determining step approximation (*K*_m_ ≫ *K*_a_) made in eq 18.

The presence of an activation barrier for lateral CO adsorption may seem surprising, considering that the cobalt atoms are in-plane with the carbon atoms of graphene. However, the stable chemisorption geometry of CO is very well-defined, with the molecule being vertically coordinated to the metal via the C atom, as shown by DFT.^19^ Similarly to the case of CO adsorbing on extended metal surfaces,^36^ the main contribution to the bond stability arises from electron backdonation from the Co 3d_*xz*,*yz*_ to the carbon monoxide antibonding π*. Both orbitals exhibit significant spatial anisotropy. Consequently, as the CO crosses the transition state during its reverse spillover from graphene to Co@Gr, it is likely to be sterically hindered, which would explain the observed activation barrier. On the other hand, it is unlikely that the activation barrier is due to a dipole–dipole electrostatic repulsion between the CO and the metal center, since the charge transfer between the Co and the underlying Ni metal is predicted to be too modest (+0.01 e^–^).^37^

## Conclusions

We investigate the kinetics of CO adsorption on single Co atoms embedded in a supported Gr layer by means of highly sensitive TDS and STM. The sticking probability on this model SAC is increased by 2 orders of magnitude by the presence of an additional reverse spillover adsorption channel. In this scenario, the majority of CO molecules do not attach directly from the gas phase, but they first physisorb on pristine Gr, diffuse on it, and finally reach the Co atom laterally to which they chemisorb, by overcoming an energy barrier *E*_a_ = 174 ± 2 meV. The study also provides a complete and self-consistent energy landscape describing CO desorption from and migration on pristine Gr. These results are important in the context of heterogeneous SAC and in gas sensing involving graphene layers whenever CO adsorption is involved, either as a beneficial or detrimental reaction.

## Supporting Information Available

TDS spectra on Gr/Ni(111) showing CO desorption from pristine Gr; STM image of Gr/Ni(111); numerical solution of the Redhead equation for first-order desorption; and Sniffer calibration procedure (PDF)
