# Supplementary material for: Reverse Spillover Dominating CO Adsorption on Single Cobalt Atoms in Graphene Divacancies
Source: J Phys Chem C Nanomater Interfaces. 2024 Dec 25;129(10):4915–22. doi: 10.1021/acs.jpcc.4c07088 (PMC11912467; doi:10.1021/acs.jpcc.4c07088)
Supplement: Supplementary file 1 — jp4c07088_si_001.pdf [file jp4c07088_si_001.pdf]

Supporting Information for Publication

Reverse Spillover Dominating CO Adsorption on Single Cobalt Atoms in Graphene di-Vacancies

[1]Francesco Armillotta, [1]Pardis Naderasli, [2,3,4,5]Valeria Chesnyak, and [1]Harald Brune**

[1]Ecole Polytechnique Fédérale de Lausanne (EPFL)Station 3, CH-1015 Lausanne, Switzerland

[2]Physics Department, University of Trieste, via A.Valerio 2, Trieste 34127

[3]CNR-Istituto Officina dei Materiali (IOM), Strada Statale 14, km 163.5, 34129 Trieste, Italy

[4]Present address: School of Chemical, Biological, and Environmental Engineering, Oregon State University, Corvallis, OR 97331, USA

[5] Present address: Physical and Computational Sciences Directorate and Institute for Integrated Catalysis, Pacific Northwest National Laboratory, Richland, WA 99354, USA

KEYWORDS: Single Atom Catalysis, SAC, cobalt, carbon monoxide, CO, adsorption, kinetics, heterogeneous catalysis, graphene

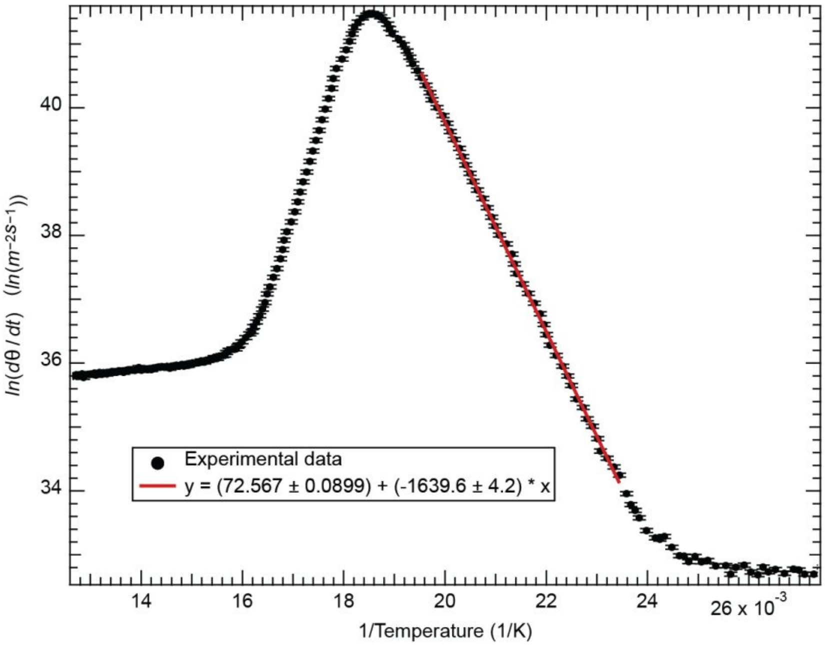

Figure S1. CO TDS spectrum of the Gr/Ni(111) sample acquired after dosing 1.0 L of CO at 30 K (heating rate 1 K/s). The desorption peak is attributed to CO desorption from pristine Gr. In the graph we plot the natural logarithm of the desorption rate ($\ln(d\theta/dt)$) as a function of $1/T$. From the linear fit (red line), performed in the low-temperature part the peak, it is possible to extrapolate the attempt frequency ($\nu_{0,a}$) and desorption energy (E_d): $\ln\left(\frac{d\theta}{dt}\right) = \ln\left(\nu_{0,d}\theta^0\right) - \frac{E_d}{k_B}\left(\frac{1}{T}\right)$. θ^0 is the CO coverage on pristine Gr at $t = 0$. $E_d = (141 \pm 1)$ meV; $\nu_{0,d} = (7.1 \pm 0.4) \times 10^{12}$ Hz

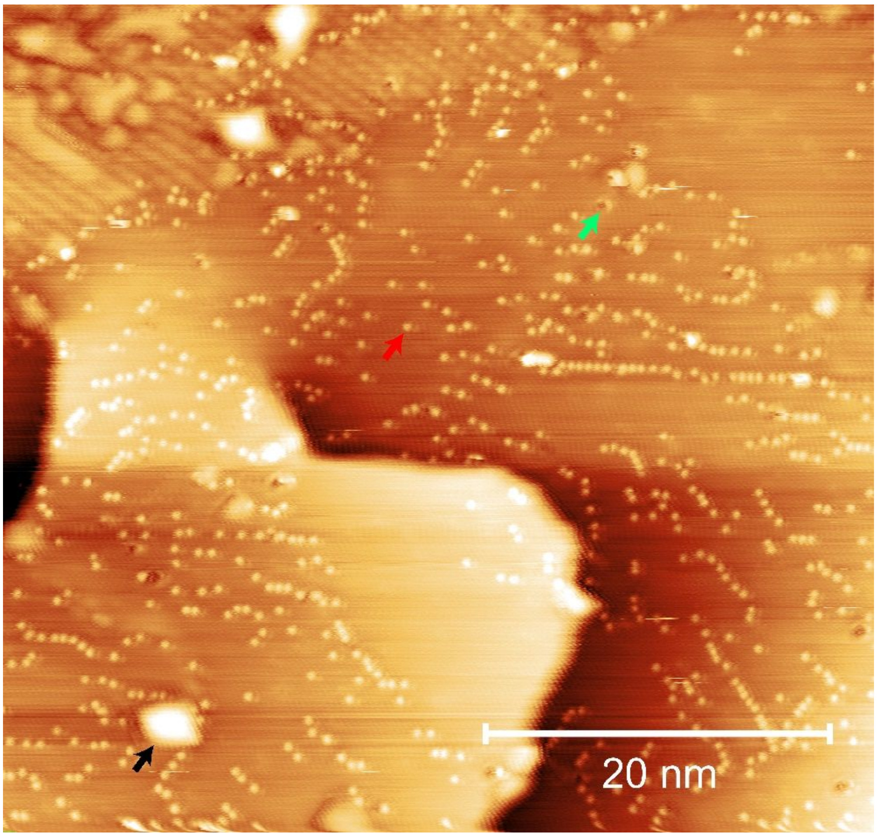

Figure S2. : Room temperature STM image representative of the Gr/Ni(111) sample. Both aligned (center-bottom) and rotated domains (top left) are visible, together with Ni@Gr (red arrow, 1.6 % ML) and dark depression (green arrow), attributed to graphene carbon vacancies. Bigger protrusions (black arrow) are associated to Gr bubbles. In rotated domains, the lower Gr-Ni interaction allows carbon segregation from the bulk. The formation of Ni_2C at the interface lifts the Gr sheet, which becomes almost freestanding.[1,2]

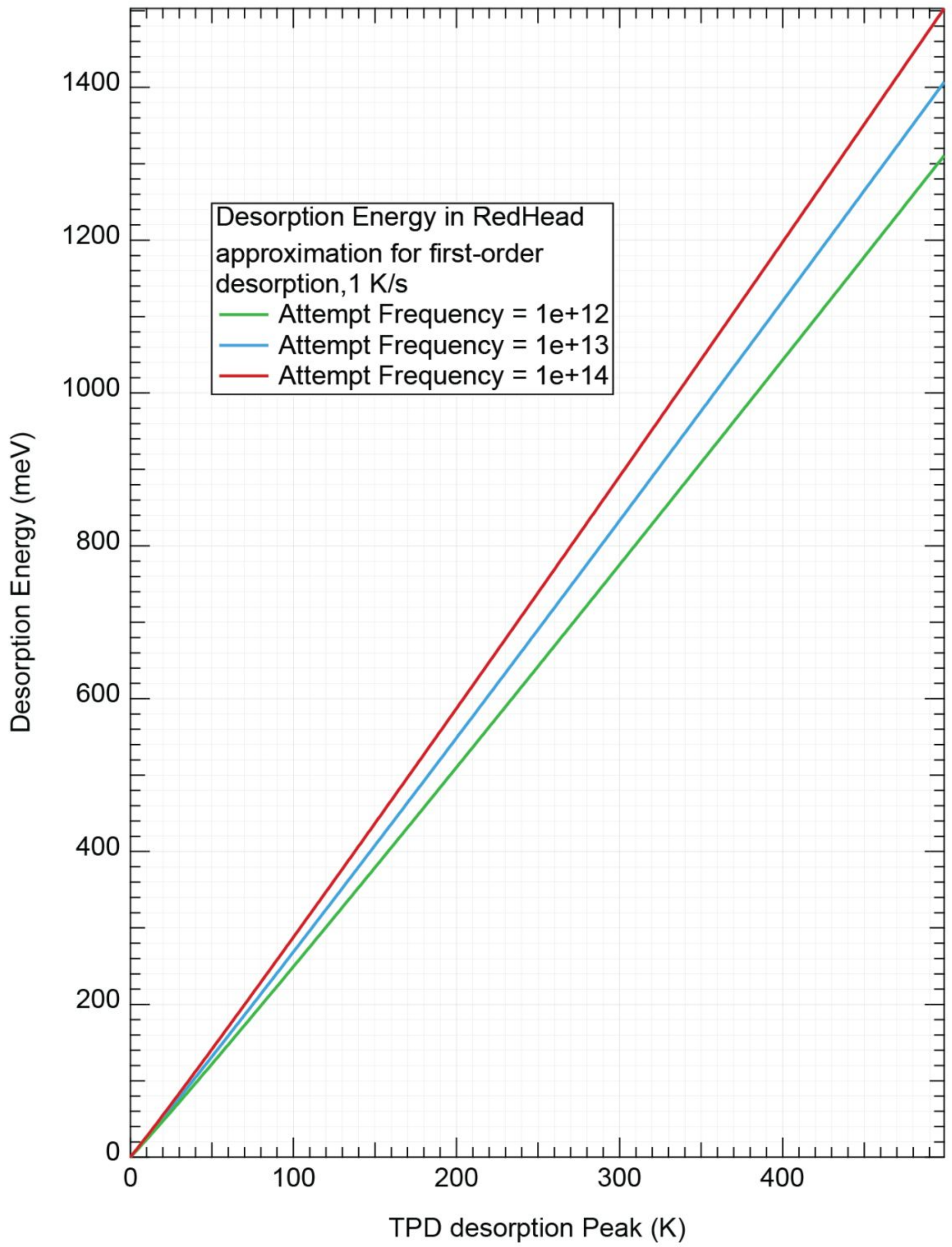

Figure S3. : The graph shows the relationship between the temperature of a first order desorption peak (T_m) in a TDS experiment (i.e., corresponding to the maximum desorption rate) and the desorption energy (E) determined by solving the Readhead approximation given below.[3] This solution is obtained numerically, for a heating rate of β =1 K/s, pre-exponential factors of ν =10^{12},10^{13}, and 10^{14} Hz, and assuming the desorption energy and pre-exponential factor being independent of coverage.

Redhead approximation:

$$\frac{E}{RT_m^2} = \frac{\nu}{\beta} e^{-E/RT_m}$$

Supporting Discussion 1: Sniffer Calibration

In this section, the calibration procedure for the Sniffer is described, for both gas dosing and TDS-spectra-to-coverage conversion. These procedures depend on the sample–Sniffer nose distance, which, for the experiments reported in this manuscript, is fixed to 100±5 µm.

For a TDS experiment, the conversion of the ion current, as read by the QMS, into absolute coverage is conducted as follows:

i. The clean surface of Ni(111) is saturated by dosing approximately 10 L CO at 30 K;
ii. The integral of the desorption profile has been set equal to 0.57 ML, according to previous studies.[4]

Once the above procedure is completed, to calibrate the amount of CO exposure the following procedure has been followed:

1. A primary calibration is performed by closing the Sniffer pumping and introducing a certain pressure of Ar inside the main chamber. This ensures that the pressure in the latter (read by a hot-filament ionization gauge) is the same as in the Sniffer's QMS;
2. With clean Ni(111) in the measurement position, 0.1 L of CO are nominally dosed, according to the calibration in step 1.;
3. The TDS area is converted to absolute coverage based on the calibration described in step ii.;
4. By using the low coverage approximation of the Langmuir equation: $\theta = \frac{DS_0}{\sqrt{2\pi m_{CO} k_B T_{CO}}}$ ($D =$ CO dosage in Pa·s) and assuming unitary sticking probability ($S_0 = 1$), the actual CO dosage can be derived and compared with the nominal 0.1 L.
5. The correction factor is applied to calibration of step 1.

REFERENCES

(1) Africh, C.; Cepek, C.; Patera, L. L.; Zamborlini, G.; Genoni, P.; Menteş, T. O.; Sala, A.; Locatelli, A.; Comelli, G. Switchable Graphene-Substrate Coupling through Formation/Dissolution of an Intercalated Ni-Carbide Layer. *Sci Rep* **2016**, *6* (1), 19734. https://doi.org/10.1038/srep19734.
(2) Africh, C.; Peressi, M.; Comelli, G. Graphene on Ni Surfaces: A Personal Journey. *Surface Science* **2025**, *753*, 122652. https://doi.org/10.1016/j.susc.2024.122652.
(3) Redhead, P. A. Thermal Desorption of Gases. *Vacuum* **1962**, *12* (4), 203–211. https://doi.org/10.1016/0042-207X(62)90978-8.
(4) Zhi Xu; Surnev, L.; Uram, K. J.; Yates, J. T. Interactions between Chemisorbed CO and Oxygen on Ni(111). *Surface Science* **1993**, *292* (3), 235–247. https://doi.org/10.1016/0039-6028(93)90329-I.
